# Risk Factors of Duodenobiliary Reflux-Related Dysfunction of Covered Biliary Metal Stents after Treatment of Duodenal Stricture in Patients with Malignant Biliary and Duodenal Obstruction

**DOI:** 10.3390/curroncol28050319

**Published:** 2021-09-26

**Authors:** Chi-Huan Wu, Mu-Hsien Lee, Yung-Kuan Tsou, Cheng-Hui Lin, Kai-Feng Sung, Kuang-Tse Pan, Nai-Jen Liu

**Affiliations:** 1 Department of Gastroenterology and Hepatology, Linkou Chang Gung Memorial Hospital, Taoyuan 333, Taiwan; s19821737@gmail.com (C.-H.W.); r5266@adm.cgmh.org.tw (M.-H.L.); flying@adm.cgmh.org.tw (Y.-K.T.); linchehui@adm.cgmh.org.tw (C.-H.L.); h12153@adm.cgmh.org.tw (K.-F.S.); 2 Department of Medical Imaging and Intervention, Linkou Chang Gung Memorial Hospital, Taoyuan 333, Taiwan

**Keywords:** duodenobiliary reflux, duodenal obstruction, recurrent biliary metal stent

## Abstract

Duodenal obstruction is often accompanied with unresectable malignant distal biliary obstruction in patients who have undergone biliary self-expandable metal stent (SEMS) placement. Duodenobiliary reflux (DBR) is a major cause of recurrent biliary obstruction (RBO) after covered biliary SEMS placement. We analyzed the risk factors for DBR-related SEMS dysfunction following treatment for malignant duodenal obstruction. Sixty-one patients with covered SEMS who underwent treatment for duodenal obstruction were included. We excluded patients with tumor-related stent dysfunction (*n* = 6) or metal stent migration (*n* = 1). Fifty-four patients who underwent covered biliary SEMS placement followed by duodenal metal stenting or surgical gastrojejunostomy were included. Eleven patients had DBR-related biliary SEMS dysfunction after treatment of duodenal obstruction. There was no difference between the duodenal metal stenting group and the surgical gastrojejunostomy group. Duodenal obstruction below the papilla of Vater and a score of ≤2 on the Gastric Outlet Obstruction Scoring System after treatment for duodenal obstruction were associated with DBR-related covered biliary SEMS dysfunction. Thus, creating a reliable route for ensuring good oral intake and avoiding DBR in patients with duodenal obstruction below the papilla of Vater are both important factors in preventing DBR-related covered biliary SEMS dysfunction.

## 1. Introduction

Patients with malignant tumors in the periampullary area have an increased risk of dual obstruction in the distal bile duct and duodenum, both of which are considered severe co-morbidities. These may be caused by several types of malignant diseases, including local, regional, or metastatic cancer. As these patients are often diagnosed at an advanced stage, efficient and effective treatment of both malignant biliary and duodenal obstruction is difficult. However, duodenal obstruction can be resolved by either duodenal self-expandable metal stent (SEMS) placement or surgical gastrojejunostomy. Biliary SEMSs have already been proven to reduce the risk of recurrent biliary strictures and the need for repeated endoscopy [1,2]. Covered biliary SEMS placement (as compared with uncovered biliary SEMS replacement) could reduce the risk of recurrent stent obstruction due to tumor ingrowth [3,4,5]. Although the possibility of tumor-related recurrent biliary obstruction (RBO) decreases after the use of covered biliary SEMS, another mechanism known as duodenobiliary reflux (DBR) serves as a major cause of RBO [6]. DBR is defined as reflux of duodenal contents, including intestinal juice, into the biliary system, which leads to RBO by biliary sludge or food particles. This phenomenon is commonly observed in patients with biliary SEMS placed across the papilla of Vater [7,8]. The literature shows that duodenal invasion is a risk factor for early dysfunction of biliary metal stents [9]. Duodenal obstruction could increase the pressure of the duodenal lumen and may aggravate DBR. Ideally, DBR-related RBO may decrease after treatment for malignant duodenal obstruction. However, some patients encountered DBR after treatment for duodenal obstruction. Thus, we aimed to analyze the risk factors of DBR-related covered biliary SEMS dysfunction after treatment for duodenal obstruction.

## 2. Materials and Methods

### 2.1. Study Design

This study was conducted as a single-center retrospective analysis and was approved by the institutional review board of our institution. Data acquisition and analysis were performed in accordance with the institutional guidelines and regulations. Owing to the retrospective design of the study, the requirement for obtaining informed consent from the patients was waived by the ethics committee.

### 2.2. Data Collection

The inclusion criteria for this study comprised patients who received covered biliary SEMS for malignant distal bile duct obstruction and underwent duodenal SEMS placement or surgical gastrojejunostomy for malignant duodenal obstruction between January 2008 and December 2020. We included patients who received covered biliary SEMS across the major papilla and consequently underwent treatment for duodenal obstruction. RBO was defined as the initial clinical success of biliary stents with subsequent typical symptoms of bile duct obstruction, such as fever or abnormal liver function tests, during a follow-up. The exclusion criteria included RBO complications due to tumor ingrowth, overgrowth, or stent migration. DBR-related SEMS dysfunction was diagnosed as the absence of enhanced tissue around the metal stent, as shown on a computed tomography scan, and no new stricture was observed on cholangiography when patients had symptoms of RBO. Duodenal obstruction was classified by location relative to the papilla: duodenal obstruction above the papilla (duodenal obstruction from the bulb to the papilla of Vater itself) and duodenal obstruction below the papilla (duodenal obstruction below the papilla, including the lower part of the second section of the duodenum to the proximal jejunum). All data, including endoscopic and radiological reports, were collected from the patients’ medical records, which included patient characteristics, primary tumor characteristics, duodenal stenosis sites, route for biliary stenting, treatment methods of duodenal obstruction, and type of covered SEMS. Outcomes were measured according to the rate of DBR-related SEMS dysfunction and SEMS patency until DBR-related SEMS dysfunction.

### 2.3. Endoscopic and Percutaneous Biliary Drainage Procedure

All biliary SEMS were deployed across the papilla of Vater. Endoscopic retrograde cholangiopancreatography (ERCP) was performed for retrograde placement of the biliary SEMS. If the ERCP procedure failed, percutaneous transhepatic biliary drainage (PTBD) for antegrade stenting was performed. Endoscopic procedures were performed using a standard duodenoscope (TJF 260 or JF 260; Olympus Optical Co., Ltd., Tokyo, Japan). Fully covered biliary SEMS (10 mm in diameter) from Bonastent (Standard Sci-Tech Inc., Seoul, Korea) and partially covered SEMSs (10 mm in diameter) from Wallstent (Boston Scientific, Marlborough, MA, USA) were used after endoscopic sphincterotomy. In cases with percutaneous biliary drainage, a 10 mm partially covered metal stent from Bonastent was inserted one week after the initial PTBD creation. Time to DBR-related biliary SEMS dysfunction was defined as the period between SEMS placement and DBR-related biliary SEMS dysfunction, or the period between SEMS placement and death if DBR-related biliary SEMS dysfunction was not observed until death.

### 2.4. Duodenal Metal Stenting and Surgical Gastrojejunostomy

The decision to treat duodenal obstruction was based on a thorough discussion between the doctors and patients. Uncovered metal stents such as BonaStent and WallFlex Enteral Stent were used for patients who chose metal stents. Surgical bypass was performed with open or laparoscopic gastrojejunostomy under general anesthesia. The type of surgery, based on the tumor location and the patient’s general condition, was decided by the surgeon. The clinical success of the treatment was defined as symptomatic relief and was measured by improvement in the standardized gastric outlet obstruction scoring system (GOOSS) score (0, no oral intake; 1, liquids only; 2, soft solids; and 3, low-residue or full diet) [10].

### 2.5. Statistical Analysis

Continuous variables are presented as mean ± standard deviation and categorical variables as frequencies and percentages. Categorical variables were analyzed using Fisher’s exact test, and a t-test was used for continuous variables. Potential risk factors for DBR-related SEMS dysfunction were analyzed using a logistic regression model. Odds ratios (ORs) and 95% confidence intervals (CIs) were calculated for each factor. Statistical significance was set at *p* < 0.05. Time to DBR-related biliary SEMS dysfunction was estimated using the Kaplan–Meier method and compared using the log-rank test. Statistical significance was set at a two-sided *p*-value of <0.05. All statistical analyses were performed using IBM SPSS Statistics version 25 (Armonk, NY, USA).

## 3. Results

### 3.1. Patients’ Characteristics

A total of 61 patients with covered biliary SEMS who underwent treatment for duodenal obstruction were recruited for the study. Patients with tumor-related RBO (*n* = 6) or metal stent migration (*n* = 1) were excluded. Fifty-four patients who underwent covered biliary SEMS placement followed by duodenal metal stenting or surgical gastrojejunostomy were included and their data were retrospectively collected. Pancreatic cancer contributed to more than half of the cases of concomitant biliary and duodenal obstruction. Most biliary SEMSs were placed endoscopically (*n* = 46, 85%). Fully covered biliary SEMSs were used in 30 patients, while 24 patients received partially covered biliary SEMSs. Duodenal obstruction above and below the papilla of Vater occurred in 36 and 18 patients, respectively. Among the 54 patients with covered biliary SEMS accompanied by duodenal obstruction, 41 patients underwent duodenal SEMS placement and 13 patients underwent surgical gastrojejunostomy. Endoscopic SEMS placement took short procedure duration and led early oral intake compared with surgical gastrojejunostomy in our series (Table 1).

### 3.2. DBR-Related Biliary SEMS Dysfunction

DBR-related SEMS dysfunction was observed in 11 patients (20.3%) with 10 events involving early dysfunction (dysfunction occurring within 3 months of SEMS placement [9]). The baseline characteristics of patients with or without DBR-related biliary SEMS dysfunction are shown in Table 2. Age, sex, and tumor stage were comparable between the two groups. The percentage of DBR-related SEMS dysfunction was similar between the two groups in terms of the type of biliary SEMS, route of biliary SEMS placement, and method for treating duodenal obstruction. However, the group with DBR-related biliary SEMS dysfunction had a significantly higher percentage of patients with duodenal obstruction below the papilla of Vater than those without DBR-related biliary SEMS dysfunction (63.63% versus 25.58%; *p* = 0.029). Following treatment for duodenal obstruction, patients with DBR-related SEMS dysfunction had a GOOSS score ≤2 more frequently than those without DBR-related SEMS dysfunction (90.90% versus 47.83%; *p* = 0.016).

### 3.3. Risk Factors of DBR-Related SEMS Dysfunction

**Figure 1:**
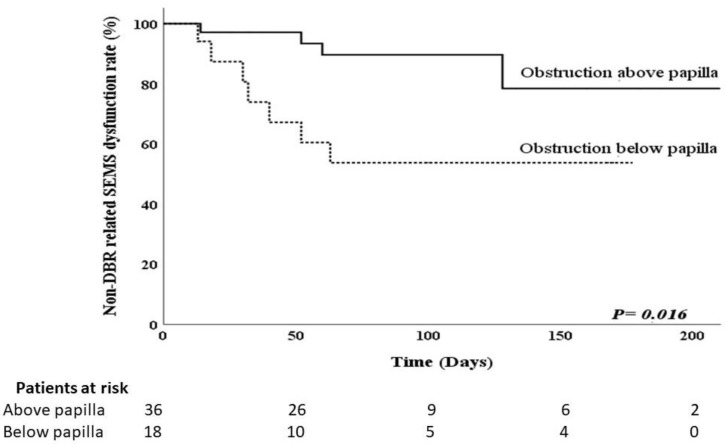
Kaplan–Meier curve showing time to duodenobiliary reflux (DBR)-related covered biliary self-expandable metal stents (SEMS) dysfunction according to the obstruction site of the duodenum.

**Figure 2:**
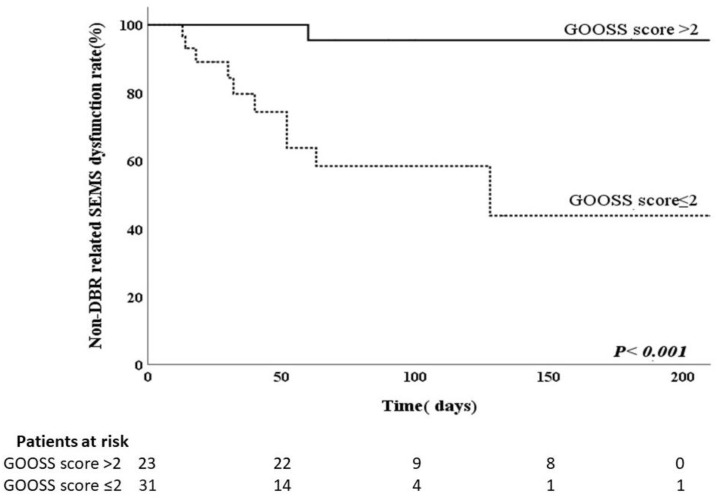
Kaplan–Meier curve showing time to duodenobiliary reflux (DBR)-related covered biliary self-expandable metal stents (SEMS) dysfunction according to gastric outlet obstruction scoring system (GOOSS) after treatment for duodenal obstruction.

The results of the analyses to identify risk factors for DBR-related SEMS dysfunction are shown in Table 3. Potential risk factors for DBR-related SEMS dysfunction include route of biliary SEMS, type of SEMS, location of duodenal obstruction, method of duodenal intervention, and post-procedure GOOSS score. These potential risk factors were further analyzed using logistic regression. Significant predictors of DBR-related SEMS dysfunction based on the univariate analysis were duodenal obstruction below the papilla of Vater and GOOSS score ≤2 after treatment for duodenal obstruction. The same results were obtained using Fisher’s exact test (Table 2). Multivariate analysis using the logistic regression model showed that duodenal obstruction below the papilla of Vater (OR, 5.984; 95% CI, 1.265–27.459; *p* = 0.024) and a GOOSS score ≤2 after treatment for duodenal obstruction (OR, 12.000; 95% CI, 1.301–110.720; *p* = 0.028) had greater odds of predicting DBR-related biliary SEMS dysfunction. We used a Kaplan–Meier curve to estimate the cumulative time to DBR-related SEMS dysfunction. The median time to DBR-related SEMS dysfunction was significantly shorter in patients with duodenal obstruction below the papilla of Vater (57.5 days) than in patients with duodenal obstruction above the papilla of Vater (90 days) (*p* = 0.024) (Figure 1). The median time to DBR-related SEMS dysfunction was significantly shorter with GOOSS score ≤2 after treatment for duodenal obstruction (38 days) than with GOOSS score >2 after treatment for duodenal obstruction (98 days) (*p* = 0.028) (Figure 2). Both factors revealed significant differences according to the log-rank test.

## 4. Discussion

**Figure 3:**
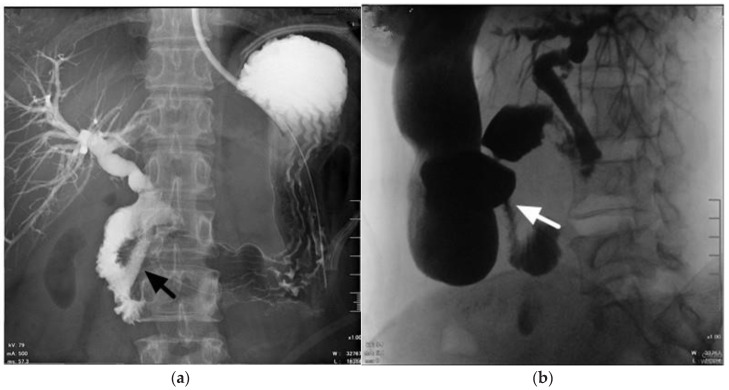
A 59-year-old woman with pancreatic cancer with concomitant distal bile duct and duodenal obstruction. The contrast medium reflux into biliary self-expandable metal stents was still observed after surgical gastrojejunostomy (black arrow) (**a**). Contrast medium passes smoothly through the gastrojejunostomy (white arrow) (**b**).

Synchronous malignant bilioduodenal obstruction is a challenging clinical scenario and a severe co-morbidity, usually caused by different malignancies such as advanced local tumors, including pancreatic cancer, bile duct cancer, or metastases that involve the peri-duodenal area [11]. Uemura et al. reported that 70% of patients with malignant duodenal obstruction developed bile duct obstruction [12]. Prolonged malignant biliary obstruction can cause cholangitis and liver failure, and malignant duodenal obstruction leads to poor oral intake and vomiting [13]. Surgical gastrojejunostomy with duodenal bypass has proven to be an effective treatment in patients with malignant duodenal obstruction. Apart from gastrojejunostomy, duodenal SEMS placement can be used for palliation of malignant duodenal obstruction [14,15,16,17]. Similar to previous trials, our series also supports that endoscopic SEMS placement has the benefits of short procedure duration and early oral intake [18,19]. Hamada et al. reported that placement of duodenal SEMSs is a risk factor for the dysfunction of a biliary SEMS and is likely caused by an increased risk of DBR [20]. However, we also experienced DBR-related SEMS dysfunction after surgical gastrojejunostomy for duodenal bypass (Figure 3). There was no difference in DBR-related SEMS dysfunction between duodenal SEMS placement and gastrojejunostomy in this study. Matsumoto et al. also reported that poor duodenal SEMS function was a risk factor for biliary stent dysfunction [21]. As mentioned above, we suggest that the actual factors that influence DBR-related SEMS dysfunction may be a function of duodenal SEMSs and gastrojejunostomy.

Mutignani et al. first described three types of duodenal strictures based on their relation to the papilla of Vater [22]. Type I stenosis occurs at the level of the duodenal bulb or upper duodenal genu and without the involvement of the papilla. Type II stenosis affects the second section of the duodenum, with involvement of the papilla. Type III stenosis involves the third section of the duodenum, distal to, and without involvement of the papilla. We simplified the position of duodenal obstruction into two categories, namely the above-papilla type (comprising patients with duodenal obstruction from the bulb to the papilla of Vater itself) and the below-papilla type (comprising patients with duodenal obstruction below the papilla, including the lower part of the second section of the duodenum to the proximal jejunum) owing to different clinical backgrounds and technical challenges [23]. Logistic regression analysis determined that the events associated with duodenal obstruction below the papilla of Vater (type III in Mutignani’s classification) were risk factors for DBR-related covered SEMS dysfunction. Hamada et al. reported endoscopic management of distal malignant biliary obstruction combined with duodenal obstruction, which included biliary drainage with a variate method of including ERCP, EUS-guided choledocoduodenostomy (EUS-CDS), or EUS-guided hepaticogastrostomy, with biliary SEMS or biliary plastic stents [24]. Recurrent biliary obstruction did not differ significantly according to the original location of duodenal obstacle. However, in this study, all the recurrent biliary obstructions that occurred in patients with the original duodenal obstruction below the papilla of Vater were due to non-tumor-related reasons, which revealed that DBR-related stent dysfunction still plays a significant role in RBO in duodenal obstruction below the papilla of Vater. Matsumoto et al. reported double-metal stenting in malignant biliary and duodenal obstruction, and included bile duct diversion strategies with biliary metal stents or biliary plastic stents [21]. In this article, we classified the position of the biliary stent’s end into two categories: above and below the duodenal stent. The rate of RBO was significantly higher when the biliary stent was above the duodenal stricture, similar to our papilla group. Owing to the high possibility of duodenal reflux in the papilla group, transmural biliary drainage such as EUS-CDS may be considered as the orifice of the biliary SEMS is away from the duodenal stricture. Anti-reflux biliary SEMS should also be considered if the patient received transpapillary drainage in the papilla group [25,26,27,28].

The study’s limitations include its retrospective, single-center design and, owing to the different types of malignancy among patients, different outcomes might have been achieved. Further studies based on prospectively collected data are needed to determine the best biliary drainage method for patients with malignant duodenal obstruction to reduce DBR-related covered biliary SEMS dysfunction.

## 5. Conclusions

In conclusion, DBR-related RBO had already been an important factor in patients who underwent covered biliary SEMS placement, especially in those with concomitant malignant duodenal obstruction. Creating a reliable route for ensuring good oral intake and avoiding DBR in patients with duodenal obstruction below the papilla of Vater are both important factors in preventing DBR-related covered biliary SEMS dysfunction.

## Figures and Tables

**Table 1:** Characteristics and outcome of patients underwent treatment of duodenal obstruction.

	Duodenal SEMS (*n* = 41)	GJJ (*n* = 13)	*p*-Value
Sex, male: female, n:n	27:14	6:7	*p* = 0.528
Age, years	66 ± 13 *	56 ± 17 *	*p* = 0.059
Pre-procedure GOOSS score	0.15 ± 0.38 *	0.41 ± 0.59 *	*p* = 0.141
Procedure time, mins	20.39 ± 6.39 *	143 ± 65 *	*p* < 0.001
Post-procedure GOOSS score	2.22 ± 0.82 *	1.85 ± 0.81 *	*p* = 0.157
Clinical success	30 (96.78%)	13 (100%)	*p* = 0.100
Day to intake, days	4.46 ± 2.31 *	12.92 ± 7.95 *	*p* < 0.001

* Values are mean ± standard deviation or number (percentage). Abbreviations: GJJ, gastrojejunostomy; SEMS, self-expandable metal stents; GOOSS, gastric outlet obstruction scoring system.

**Table 2:** Characteristics and outcome of patients with or without duodenobiliary reflux-related covered biliary self-expandable metal stent dysfunction after treatment of duodenal obstruction.

	Dysfunction (+) (*n* = 11)	Dysfunction (-) (*n* = 43)	*p*-Value
Sex, male:female, n:n	7:4	23:20	*p* = 0.546
Age, years	60 ± 15 *	65 ± 13 *	*p* = 0.331
TNM stage, III:IV, n:n	1:10	11:32	*p* = 0.424
Underlying malignancy			
Pancreatic cancer	6 (54.55%)	33 (76.74%)	
Duodenal/ampullary cancer	1 (16.67%)	4 (9.30%)	
Metastatic cancers	4 (36.36%)	2 (4.65%)	
Bile duct cancer	0 (0.00%)	4 (9.30%)	
Biliary SEMS			
ERCP/PTBD	10:1	36:7	*p* = 0.100
Fully covered/Partially covered	9:2	21:22	*p* = 0.087
Treatment for duodenal obstruction			
Stricture above/Below papilla	4:7	32:11	*p* = 0.029
Duodenal SEMS: GJJ	6:5	35:8	*p* = 0.149
GOOSS score ≤2 after treatment for duodenal obstruction	10 (90.90%)	21 (48.83%)	*p* = 0.016
Peritoneal carcinomatosis	6 (64.55%)	29 (67.44%)	*p* = 0.489
Chemotherapy	7 (63.63%)	28 (65.11%)	*p* = 0.489

* Values are mean ± standard deviation or number (percentage). Abbreviations: ERCP, endoscopic retrograde cholangiopancreatography; GJJ, gastrojejunostomy; GOOSS, gastric outlet obstruction scoring system; PTBD, percutaneous transhepatic biliary drainage; SEMS, self-expandable metal stents; TNM, tumor/lymph node/metastasis.

**Table 3:** Risk factors for duodenobiliary reflux-related stent dysfunction after treatment of duodenal obstruction.

	Univariate OR	*p*-Value	95% CI for OR	Multivariate OR	*p*-Value	95% CI for OR
Variate						
Route of biliary SEMSs						
ERCP	1.994					
PTCD	Reference	0.555	0.213–17.713			
Type of the SEMS						
Fully covered	4.714					
Partially covered	Reference	0.065	0.910–24.418			
Location of duodenal obstruction						
Below the papilla of Vater	5.091					
Above the papilla of Vater	Reference	0.023	1.247–20.781	5.894	0.024	1.265–27.459
Duodenal intervention						
GJJ	3.646					
Duodenal SEMSs	Reference	0.073	0.887–14.988			
GOOSS score after treatment for duodenal obstruction						
≤2	10.476					
>2	Reference	0.032	1.232–89.115	12.000	0.028	1.301–110.720

Abbreviations: CI, confidence interval; ERCP, endoscopic retrograde cholangiopancreatography; GJJ, gastrojejunostomy; GOOSS, gastric outlet obstruction scoring system; SEMS, self-expandable metal stents; PTBD, percutaneous transhepatic biliary drainage.

## Data Availability

The data presented in this study are available on reasonable request from the corresponding author. The data are not publicly available due to ethical concerns.
